# Practical Observations on the Use and Abuse of Cold and Warm Sea-Bathing in Various Diseases, Particularly in Scrofulous and Gouty Cases

**Published:** 1813-09

**Authors:** 


### CRITICAL ANALYSIS
### *OF RECENT PUBLICATIONS*
IN THE
### DIFFERENT BRANCHES OF PHYSIC, SURGERY, AND MEDICAL PHILOSOPHY.

*Practical Observations on the Use and Abuse of cold and warm Sea-bathing in various Diseases, particularly in Scrofulous and Gouty Cases. By* John Gibney, M.D. Resident Physician at Brighton. 8vo. pp. 144. Underwood. 1813.

HACKNEYED as the subject of this treatise is, the author has contrived to render it interesting. He has brought together numerous authorities, and adduced some original remarks from his own experience. If well-informed medical men will not derive much instruction from the volume, we think they will find nothing in it to which they can object; whilst young practitioners, and those not much in the habit of thinking for themselves, will undoubtedly meet with new and instructive matter. Invalids, also, will do well to read the book; for they will find that sea-bathing, improperly pursued, is as capable of doing mischief, as under skilful regulation it may be productive of decided benefit. It unquestionably is an important agent, and, in many cases, requires nice discrimination, both in the mode and time of its application. The temperature of the bath, in all cases, should be determined by the physician.

Dr. Gibney's cautions respecting the use of the cold bath in advanced age and in infancy, are very judicious. Few, he observes, in the evening of life, are benefited by it; and many young delicate children suffer from its use, although it has been absurdly considered as strengthening and invigorating the system. The intermediate age is stated to be most favorable for cold bathing.

The following is an instructive case:

"In November last, a person of extensive commercial connections, aged 68, became extremely irritable and unhappy, in consequence of pecuniary losses; he passed sleepless nights, and his appetite forsook him. On coming to Brighton, he imprudently entered into a warm bath of 100°, while his intestines were constipated, his pulse high and quick, and his heat at night very considerable. The consequence was, he became feverish, his head affected with a fixed pain, and all his uncomfortable feelings considerably increased. He was about to abandon the warm bath in despair; but on using purgative medicines, taking his bath at 92° in the forenoon, avoiding wine and cordial medicines, medicines, of which he had used great quantities, his general health was soon reinstated. His error consisted in the abuse of a useful, and to him salutary remedy; having employed it at an improper time, and of a temperature unsuitable to the nature and state of his disease."

Dr. Gibney notices the trifling manner in which the warm bath is too generally used in this country; many persons going into it only once or twice a-week, and remaining immersed a very short time.

"In Switzerland, the time of remaining in the warm bath is from six to twelve hours. At Pfeffers, one half of the body is exposed for many hours in succession to warm vapor, while the other half is immersed in the bath. At Landeck, in Silesia, the practice of continuing in the warm baths, in all cases of debility, for hours at a time, is also very general, and attended with the happiest effects."

From these and similar facts, the author demonstrates the absurdity of the opinion which pronounces all warm baths to be relaxing. Great misapprehension on this subject has long possessed the vulgar mind in this country, probably from the people not being familiar with the extensive use and pleasant effects of baths as practised in most countries of Europe and the East. Indeed, it is much to be lamented, that in the neighbourhood of most large towns in England, bathing of all sorts is extremely discouraged; for, though it is always desirable to have recourse to sea-bathing, this luxury can only be enjoyed by a very small portion of society, and river-bathing is better than none. If the people were duly impressed with the value of even fresh-water immersion, both warm and cold, the means of obtaining it would not long be wanting. There is no pleasure, and but little advantage, in resorting to the close ill-ventilated baths of the metropolis; we want something upon a larger scale, and a more liberal principle. Is the Thames smaller, or more inaccessible, than the Seine, that we cannot have our swimming schools? Is the science of hydraulics less understood now than in the age of Nero, that we cannot enjoy our *Thermæ Marinæ* in the heart of London?

Dr. Gibney has collected many facts to prove that heat is not relaxing, as is too generally imagined. Duly modified, it "gives renovated vigor to animal and vegetable existence, and only produces relaxation when immoderately applied." This is most strongly exemplified in those warm climates where it is not excessive; there, the inhabitants are accustomed to remain long in the warm bath, which, as they express it, "feeds and nourishes their blood." Were it relaxing, the relief it is known to afford in cases of the utmost debility, attended with colliquative sweats, would not necessarily follow. In the Levant, and in Italy, no disease of relaxation is thought to be effectually removed, without the warm bath; and in every species of intermittent, where affection of the liver, &c. often accompany the disease, its successful application is remarkable. In Spain, a kind of vapor bath, of a very efficacious and penetrating nature, is formed from the pulp of olives, the *Horeijo de Areitunas*, after the oil is expressed, by suffering a gradual fermentation to take place; when by this process the necessary heat is generated, the patient is confined under the heap, a due attention being paid to the position, so as that respiration may be free; and by remaining for one, two, or three hours, and repeating the bath at regular periods for many days in succession, several diseases arising from debility are most effectually removed.

"In this country (Dr. Gibney observes) I have known brewers grains, while possessing the proper degree of heat, serve purposes very similar; as in debility of the spine, and in some stages of that dreadful strumous disease which so often affects the hip-joint: in cases like these, heat, whether applied in the form of vapor or otherwise, gives vigorous tone to the action of the skin, that important emunctory, on whose healthy condition our nervous energy, the digestive powers, the secretory functions of our vital organs, and the regular circulation of our blood, depend in so considerable a degree; and which, when at all discomposed, is restored to order by the warm bath, much more certainly and effectually than by any other means with which we are acquainted. In cases of suspended animation from drowning, inhaling noxious air, or from any other cause, the revivifying power of heat is well known; but except the application of it, in these and other cases, be continued for a considerable length of time, it will often fail to produce the effects expected."

The proper time for entering the warm bath has not always been sufficiently observed. Dr. Gibney's remarks on this subject merit attention. The work concludes with a brief account of those diseases in which the bath is applicable; and on the whole we consider it a very creditable performance. We commend it too the more cheerfully, because the author has not spoken of the remedy more highly than it deserves, and has not augmented the size of the volume by inserting long and tedious cases.

